# Enhancing Stallion Semen Cryopreservation: Selected Antioxidant Extracts and Sperm Freezability

**DOI:** 10.3390/antiox14111363

**Published:** 2025-11-16

**Authors:** Raffaele Boni, Raffaella Ruggiero, Felisia De Luca, Graziano Preziosi, Maria Antonietta Ferrara, Angela Ostuni, Simone Guerriero, Alessandra Gallo, Carola Murano, Stefano Cecchini Gualandi

**Affiliations:** 1 Department of Basic and Applied Sciences, University of Basilicata, Via dell’Ateneo Lucano, 10, 85100 Potenza, Italyfelisia.deluca@studenti.unibas.it (F.D.L.); angela.ostuni@unibas.it (A.O.); 2 Department of Biology and Evolution of Marine Organisms, Stazione Zoologica Anton Dohrn, Villa Comunale, 80121 Naples, Italy; simone.guerriero@szn.it (S.G.); alessandra.gallo@szn.it (A.G.); 3 Unit of Naples, Institute of Applied Sciences and Intelligent Systems, Italian National Research Council (ISASI-CNR), Via Pietro Castellino 111, 80131 Napoli, Italy; graziano.preziosi@na.isasi.cnr.it (G.P.); antonella.ferrara@na.isasi.cnr.it (M.A.F.); 4 Department of Integrative Marine Ecology, Stazione Zoologica Anton Dohrn, Villa Comunale, 80121 Naples, Italy

**Keywords:** stallion sperm, stallion age, sperm freezing, antioxidant extracts, matcha, spirulina, horseradish, quercetin

## Abstract

Cryopreservation of equine semen remains challenging due to pronounced individual variability in cryotolerance. Because freezing induces oxidative stress and spermatozoa are particularly susceptible to such damage, this study aimed to comparatively evaluate the effects of natural extracts from nutraceutical compounds with high antioxidant activity, specifically matcha, spirulina, and horseradish, as well as quercetin, a well-known antioxidant molecule. These compounds were added to the freezing extender, and semen from 12 Salernitano stallions (48 ejaculates in total) was analyzed. Several parameters were assessed, including sperm kinetics, bioenergetics, oxidative and nitrosative stress markers, and the sperm DNA fragmentation index, both before and after cryopreservation. Neither the natural extracts nor quercetin significantly improved sperm freezability, likely due to the high degree of inter-individual variability. Stallion age also had a significant effect on nearly all the parameters evaluated, although no significant interactions were observed between age and treatment for any of the sperm quality traits. In conclusion, supplementation of the freezing extender with matcha, spirulina, horseradish extracts, or quercetin did not significantly enhance stallion semen cryopreservation outcomes. Conversely, stallion age and individual variability had a marked effect on sperm cryotolerance, highlighting the need for customized and holistic strategies to optimize cryotolerance in individual stallions.

## 1. Introduction

The study of natural substances for therapeutic and preventive purposes has ancient origins but has gained renewed attention in recent years, particularly as an alternative to chemically synthesized pharmaceuticals [1]. Among the biological activities of natural compounds, antioxidant effects are especially well studied, with implications in aging, cardiovascular disease, cancer, and neurodegeneration [2,3,4]. These properties are also highly relevant to male germplasm preservation, as sperm cells are extremely vulnerable to oxidative and nitrosative stress caused by reactive oxygen and nitrogen species (ROS, RNS). Several factors contribute to this occurrence: (i) the high metabolic activity required for motility [5]; (ii) the small cytoplasmic volume, which limits intrinsic antioxidant defenses [6]; and (iii) the partial removal of seminal plasma during processing, which otherwise provides substantial antioxidant protection [7,8]. In addition, microbial contamination increases the production of H_2_O_2_ in cool-stored semen, which is associated with reduced tolerance to refrigeration. [9]. Furthermore, procedures such as cooling and cryopreservation further reduce antioxidant protection, exposing sperm to oxidative damage [10].

The main targets of ROS and RNS in sperm are lipids, DNA, and proteins [11]. Lipid peroxidation decreases membrane fluidity, impairing motility [12]. Although sperm DNA is highly condensed, it remains susceptible to oxidative fragmentation, which can compromise fertility [13,14,15]. Proteins are also affected by oxidative modifications that alter their structure and function [16].

Cryopreservation remains the only reliable method for long-term storage of male germplasm, supporting livestock breeding programs and the conservation of endangered species [17,18]. However, freezing and thawing impose additional stress through ice crystal formation, osmotic imbalance, and oxidative damage, while also remodeling sperm membrane proteins in a manner that mimics premature capacitation [19,20,21]. Sperm resilience to cryopreservation varies both across and within species [22]. This variability is less problematic in species where cryopreservation techniques are well established, such as cattle, where post-thaw sperm quality is a major criterion for selecting breeding males [23]. In horses and humans, however, selective breeding based on cryotolerance is not feasible; thus, optimized protocols and customized extenders supplemented with cryoprotective additives are required to improve sperm cryosurvival [24].

Natural compounds with nutraceutical and antioxidant properties have been widely investigated for this purpose, either as dietary supplements or as additives in semen extenders [25,26]. In this study, we tested three natural products—matcha, spirulina, and horseradish—known for their antioxidant activity, by incorporating their extracts into stallion semen freezing extenders. As a reference, quercetin, a well-characterized antioxidant previously shown to protect sperm during cryopreservation in several species, was also included [27].

Matcha, a green tea (*Camellia sinensis*) product grown under shaded conditions, is rich in catechins, theanine, and caffeine [28]. Reports on its effects in sperm cryopreservation are inconsistent, showing no significant benefit in pigs [29], but positive results in rams [30], bulls [31], and humans [32].

*Spirulina platensis* is a filamentous cyanobacterium containing proteins, vitamins, minerals, γ-linolenic acid, and pigments with strong antioxidant properties. Its defense system includes both enzymatic (superoxide dismutase, glutathione peroxidase) and non-enzymatic (carotenoids, tocopherol, chlorophyll derivatives) antioxidants [33,34]. Supplementation of freezing extenders with Spirulina extracts has been shown to improve sperm motility and viability in buffalo bulls [35]. In stallions, *Spirulina* addition has reduced cryodamage and improved post-thaw sperm quality [36].

Horseradish (*Armoracia rusticana*) is valued for its antioxidant-rich roots, which contain glucosinolates, isothiocyanates, enzymes, flavonoids, phenolic acids, vitamins, minerals, and essential oils [37,38]. To the best of our knowledge, no studies have examined the use of this plant in sperm cryopreservation. It is sometimes erroneously associated with *Moringa oleifera*, a distinct species with different nutraceutical properties and documented protective effects during sperm freezing [39].

As a reference compound, quercetin was employed. Quercetin is a plant-derived flavonol belonging to the flavonoid subclass of polyphenols and is abundantly present in various vegetables [40]. It exhibits potent antioxidant activity, suppressing intracellular superoxide ion generation, chelating iron, and inhibiting lipid peroxyl radical formation [41]. In ram sperm, quercetin has been shown to reduce motility and modulate mitochondrial activity by inhibiting plasma membrane calcium pumps, thereby prolonging sperm metabolism during storage [42]. However, its effectiveness in preventing sperm damage caused by freezing remains controversial [43].

The aim of the present study was to evaluate the effectiveness of these natural extracts in stallion semen cryopreservation, focusing on physiological parameters such as sperm kinetics, bioenergetics, oxidative and nitrosative stress markers, and DNA fragmentation. In addition, we investigated how stallion age influences sperm responses to these treatments.

## 2. Materials and Methods

### 2.1. Materials

Unless otherwise specified, all materials used in this study were purchased from Merck Life Science (Milan, Italy) and were certified as cell culture–tested.

### 2.2. Animals and Husbandry

Between February and May 2025, semen was collected weekly from 12 clinically healthy, fertile Salernitano stallions (aged 4–18 years; mean ± SD: 11.2 ± 5.9). The animals were housed individually in paddocks at the Regional Center of Equine Improvement (Caserta, Italy) under standardized conditions. The diet consisted of hay and concentrate, with free access to water and no antioxidant supplementation. The facility is licensed for equine semen collection by the Campania Region (authorization: U1500083 CE000642004) and adheres to strict health and welfare regulations. All animal procedures complied with the European Directive 2010/63/EU and Italian legislation (D. Lgs. 26/2014), ensuring minimal stress and reduced animal use.

### 2.3. Experimental Design

**Figure 1:**
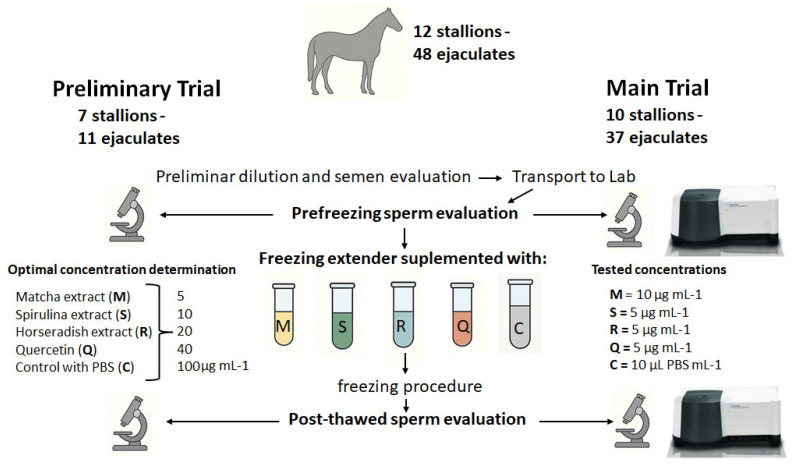
Schematic representation of the experimental design used to evaluate the cryoprotective effects of three natural extracts (matcha, spirulina, and horseradish) and quercetin on stallion sperm.

A preliminary trial was conducted to determine the optimal dosage of each extender supplement (Figure 1). This involved seven stallions, with a total of 11 ejaculates collected. Each extract powder and quercetin were dissolved in PBS at 1 mg mL^−1^ and then added individually to the freezing extender. Five treatment groups were prepared for each extract and for quercetin by adding increasing concentrations (5, 10, 20, 40, and 100 µg mL^−1^) to the HF-20 extender before sperm addition (100 × 10^6^/mL). An additional control group received PBS alone, in a volume equivalent to that used for the highest extract concentration.

Once the optimal concentrations were established, a subsequent trial was performed (Figure 1) using 10 stallions (mean ± SD: 9.8 ± 5.4 years). Except for one stallion, which provided a single ejaculate, each contributed four ejaculates throughout the study. In total, 37 semen collections were carried out in groups of two to three animals, with a new group enrolled after every fourth collection. Collections were performed weekly, and no samples were obtained outside the trial period.

Semen samples were initially evaluated for volume, motility, and concentration. They were then diluted 1:2 with INRA 96^®^ and transported under controlled cooling conditions until reaching room temperature (RT, 20 °C). Upon arrival at the laboratory, samples were further analyzed for sperm kinetics and function. Each ejaculate was divided equally into tubes containing freezing extenders supplemented with one of three natural extracts (matcha, spirulina, or horseradish). An additional group was treated with quercetin, while a control group received no supplementation.

### 2.4. Extracts of Natural Products

The aqueous extract of matcha (*Camellia sinensis*) was prepared following the method of Sayuti et al. [44] with minor modifications. Briefly, 2 g of a commercial matcha powder (International Food Europe s.r.l., Monterotondo, Rome, Italy) was infused in 100 mL of Milli-Q water at +80 °C for 15 min under gentle stirring (150 rpm). The infusion was then cooled to RT, centrifuged at 3500× *g* for 20 min at 4 °C, filtered through a 0.22 μm syringe filter, and lyophilized prior to storage at −80 °C until further use.

The extraction of Spirulina (*Arthrospira platensis*) was performed following the method of Tavakoli et al. [45] with slight modifications. Specifically, 2 g of a commercial product (Unterhofer & Partner Sas, Bolzano, Italy), containing dehydrated spirulina cells, was soaked in 100 mL of Milli-Q water at RT for 60 min under gentle stirring. The mixture was then sonicated at 30 kHz using a Sonopuls HD2070 device (Bandelin electronic GmbH & Co., Berlin, Germany), applying two cycles of 10 min each. Following sonication, the suspension was left to soak for an additional 12 h at +10 °C under gentle stirring. The resulting mixture was subsequently centrifuged and filtered, and the obtained extract was lyophilized. Dried extracts were stored at −80 °C until further use.

The aqueous extract of fresh horseradish (*Armoracia rusticana*) was prepared following the method described by Calabrone et al. [46] with slight modifications using roots harvested from field-grown plants in Southern Italy (San Chirico Raparo, Basilicata; latitude 40°11′32.712″ N, longitude 16°4′34.248″ E). Samples were collected from the central portion of the root, thoroughly rinsed with distilled water, blotted dry with paper towels, cut into small pieces, and stored at −80 °C. The frozen material was then lyophilized and ground into a fine powder. For extraction, 2 g of the lyophilized powder were placed in a conical flask with 100 mL of Milli-Q water and gently stirred at room temperature for 12 h. The resulting mixture was subjected to centrifugation and filtration, and the aqueous extract was subsequently lyophilized. The extracts were stored at −80 °C until use.

Extraction yields (*w*/*w*) were determined in triplicate as follows:

$$\mathrm{Yield}\;(\%)=\frac{\mathrm{weight}\;\mathrm{of}\;\mathrm{extract}}{\mathrm{weight}\;\mathrm{of}\;\mathrm{sample}}\times 100$$

### 2.5. Phytochemical Analyses

For the phytochemical analyses, each extract was dissolved in Milli-Q water at a concentration of 1 mg mL^−1^. Quercetin (code Q4951, Sigma-Aldrich, Milan, Italy) was also tested at the same concentration as the extracts. Ascorbic acid (AA, code A5960, Sigma-Aldrich, Milan, Italy) was used as an internal standard in some of the assays.

The total antioxidant capacity (TAC) of the extracts was assessed using the ferric reducing antioxidant power (FRAP) assay, following the procedure described by Benzie and Strain [47]. TAC values were calculated based on a calibration curve prepared with iron(II) sulfate heptahydrate at concentrations ranging from 62.5 to 1000 μM, and results were expressed as μg of iron sulfate equivalents (ISE) mg^−1^ of extract dry weight. Aqueous solutions of AA (1 mg mL^−1^) served as positive controls.

The total reducing power (TRP) of the extracts was evaluated following the protocol described by Oyaizu [48]. TRP values were calculated using a standard curve generated with AA solutions ranging from 4.69 to 300 μg mL^−1^, and results were expressed as μg of ascorbic acid equivalents (AAE) mg^−1^ of extract dry weight.

The free radical scavenging activity (FRSA) of the extracts was measured using the 1,1-diphenyl-2-picrylhydrazyl (DPPH) assay, following a modified version of the method originally proposed by Blois [49]. In this procedure, each sample was mixed with a 0.5 mM DPPH solution and incubated in the dark for 20 min before absorbance was recorded. The scavenging activity was calculated using the following formula:

Inhibition (%) = [1 − (A_s_/A_0_)] × 100

where A_s_ represents the absorbance of the sample and A_0_ the absorbance of the DPPH control. Aqueous AA solutions (1 mg mL^−1^) were included as positive controls.

The total polyphenol content (TPC) of the samples was quantified using the Folin–Ciocalteu reagent, following the procedure outlined in ISO standard 14502-1 [50]. TPC values were determined from a calibration curve prepared with gallic acid, within a concentration range of 10 to 50 μg mL^−1^, and expressed as μg of gallic acid equivalents (GAE) mg^−1^ of extract dry weight.

The total flavonoid content (TFC) was assessed using the aluminum chloride (AlCl_3_) colorimetric method, as described by Zhishen and colleagues [51], with quercetin employed as the reference compound. Quantification was based on a standard curve generated with quercetin solutions ranging from 12.5 to 400 μg mL^−1^, and results were reported as μg of quercetin equivalents (QE) mg^−1^ of extract dry weight.

### 2.6. Semen Collection and Transport

Semen was collected using a Missouri artificial vagina fitted with in-line sterile gauze to remove the gel fraction. Stallions underwent three preliminary collections before the trial to clear the epididymal reserve. The gel-free semen volume was measured, diluted 1:2 with pre-warmed INRA 96^®^, and cooled gradually to room temperature (~20 °C) over ~2 h using an insulated container with 37 °C water and external frozen eutectic plates (cooling rate: 0.10–0.15 °C min^−1^).

### 2.7. Sperm Concentration and Kinematic Evaluation

At the University of Basilicata’s Laboratory of Animal Reproduction, semen concentration and motility were determined using a Makler chamber and computer-assisted sperm analysis (CASA; SCA 5.0, Microptic, Barcelona, Spain) [52]. Samples were diluted to 30 × 10^6^ sperm mL^−1^ in INRA 96^®^ and equilibrated at 37 °C. Kinematic parameters included total motility (TM), progressive motility (PM), curvilinear velocity (VCL), straight-line velocity (VSL), and average path velocity (VAP). Motile sperm were defined as those with an average velocity > 10 µm s^−1^, and ≥1000 sperm tracks were analyzed per sample.

### 2.8. Mitochondrial Membrane Potential (MMP)

MMP was assessed using JC-1 staining, which emits green (~535 nm) or red (~595 nm) fluorescence depending on mitochondrial polarization. After washing, 1 × 10^6^ sperm cells were incubated with JC-1 (1.5 μM) at room temperature for 30 min, washed again, and analyzed using a spectrofluorometer (Cary Eclipse, Agilent, Cernusco Sul Naviglio, Milan, Italy). MMP was calculated as the fluorescence intensity ratio (J_0_B/J_0_A) between the two emission peaks [53].

### 2.9. Lipid Peroxidation (LPO)

LPO levels were measured using the fluorescent dye C11-BODIPY^581/591^, which shifts emission peaks from ~595 nm (C_0_B) to ~520 nm (C_0_A) upon oxidation. Sperm aliquots (1 × 10^6^) were incubated with the dye (2 μM) in PBS-PVA for 30 min at room temperature, washed, and analyzed using a spectrofluorometer, as above. Oxidative status was expressed as the ratio of the oxidized (C_0_A) to total fluorescence intensity (C_0_A + C_0_B) [53].

### 2.10. Intracellular Reactive Oxygen Species (ROS)

Intracellular ROS content was measured using the dye H_2_DCFDA. Sperm (1 × 10^6^ cells) were washed, stained with 10 μM H_2_DCFDA in PBS-PVA, incubated for 30 min in the dark, and then rewashed before fluorescence readings. Emission at ~525 nm (excited at 488 nm) was recorded using a spectrofluorometer, as above, to quantify intracellular ROS levels in arbitrary units [53].

### 2.11. Intracellular Nitric Oxide (NO)

NO levels were determined with DAF-FM diacetate staining. Sperm aliquots (1 × 10^6^ cells) were incubated with 10 μM DAF-FM in PBS-PVA for 30 min in the dark, washed, and read with a spectrofluorometer for fluorescence quantification. Emission was measured at ~525 nm after excitation at 488 nm [53].

### 2.12. DNA Fragmentation Index (DFI)

DNA fragmentation index (DFI) was evaluated using two complementary approaches: a direct assay, the terminal deoxynucleotidyl transferase (TdT) dUTP Nick-End Labeling (APO-BrdU TUNEL Assay Kit, A23210, Thermo Fisher Scientific Inc., Rodano, Milan, Italy), and an indirect assay, the sperm chromatin structure assay (SCSA). These two techniques provide distinct yet related information about DNA integrity. The combination of these methods offers a comprehensive view of DNA damage of sperm samples.

The TUNEL assay detects fragmented DNA by enzymatically labeling exposed 3′-OH ends with the thymidine analog 5-bromo-2′-deoxyuridine 5′-triphosphate (BrdUTP). This reaction is catalyzed by terminal deoxynucleotidyl transferase (TdT), allowing BrdUTP incorporation at the sites of both single- and double-strand DNA breaks. Incorporated BrdU residues are subsequently identified using an Alexa Fluor 488–conjugated anti-BrdU antibody [54]. For the assay, 1–2 × 10^6^ spermatozoa were fixed in 2% paraformaldehyde for 1 h, washed in PBS-PVA, and stored at −20 °C in PBS-PVA containing 1% sodium azide. Prior to labeling, cells were washed and permeabilized overnight in 70% ethanol at −20 °C. Samples were then incubated with the TUNEL reaction mixture containing TdT and BrdUTP for 60 min at 37 °C, followed by labeling with the Alexa Fluor 488-conjugated antibody in the presence of RNase A. Nuclei were counterstained with propidium iodide (PI) to visualize total DNA content. Fluorescence signals from Alexa Fluor 488 and PI were measured by fluorescent spectroscopy with a plate reader (Infinite M2000 PRO; Tecan, Männedorf, Switzerland) using excitation at 488 nm and 535 nm, with emissions recorded at 525 nm and 620 nm, respectively.

SCSA measures the susceptibility of chromatin to denaturation after acid treatment, thereby reflecting chromatin stability and packaging, and is based on the metachromatic properties of acridine orange (AO) [55]. After excitation at 488 nm, AO emits green fluorescence (~530 nm) when intercalated into intact double-stranded DNA and red fluorescence (~647 nm) when bound to denatured single-stranded DNA [55]. The original stallion semen protocol was described by Love and Kenney [56], but here it was adapted to a 96-well plate format. Briefly, 1 × 10^6^ spermatozoa were fixed in paraformaldehyde, washed, and stored as described above. For analysis, cells were resuspended in 15 µL SCSA buffer, treated with 30 µL detergent/acid solution, and after 30 s stained with 90 µL AO buffer. The resulting fluorescence patterns at the spectrofluorometer (see above) provided quantitative information on chromatin stability and susceptibility to fragmentation. DFI^SCSA^ was calculated as the ratio of the red emission peak (647 nm, F_0_R) to the sum of the red (647 nm, F_0_R) and the green (~530 nm, F_0_G) emission peaks. For both tests, positive controls were treated for 24 h with 300 and 600 µM hydrogen peroxide [57] (Figure S1).

### 2.13. Cryopreservation and Post-Thaw Analysis

Sperm samples (100 × 10^6^ mL^−1^) diluted in HF-20 extender, either supplemented or unsupplemented (control, C0), were cooled to 4 °C over 1 h in a refrigerated incubator (FOC 225D, VELP Scientifica S.r.l., Usmate Velate, Monza-Brianza, Italy). Straws were filled, sealed, equilibrated for 15 min at 4 °C, frozen 4 cm above liquid nitrogen for 10 min, and then stored in liquid nitrogen. After one week, seven straws per treatment per stallion were thawed (10 s in air, followed by 30 s at 37 °C), and their contents were centrifuged, and purified using a 60/40 Percoll gradient to remove extender residues. The final pellet was divided for motility (CASA) and other physiological assessments (MMP, LPO, ROS, NO, DFI) [53].

### 2.14. Statistical Analysis

Data were analyzed by ANOVA using Systat (v11.0; Systat Software Inc., San Jose, CA, USA). Two- and three-way ANOVAs assessed treatment, stallion, and age effects on refrigerated and frozen–thawed samples. Percentage data were arcsine-transformed before analysis. Normality and homogeneity of variance were verified using the Shapiro–Wilk and Levene tests, respectively. Pairwise mean comparisons were performed with Tukey’s test. Linear regression was used to calculate correlation coefficients (R). Partial least squares discriminant analysis (PLS-DA) was conducted using Python v3.12. A significance threshold of *p* < 0.05 was applied. Results are presented as mean ± standard deviation (SD) or mean ± standard error of the mean (SEM).

## 3. Results

### 3.1. Antioxidant Properties and Phytochemical Evaluation of the Extracts

The antioxidant and related properties of the natural product extracts under investigation, as well as those of quercetin, used as a reference compound (benchmark), are presented in Table 1. The extraction yields (on dry-weight basis) of the aqueous extracts were 27.95 ± 1.32%, 32.70 ± 1.78%, and 25.50 ± 0.97% for matcha, spirulina and horseradish, respectively. The matcha extract exhibited significantly higher TAC and TFC values compared to the other extracts and to quercetin. For the remaining evaluations, matcha showed higher values than the other extracts, but lower (TRP and TPC) or comparable values relative to quercetin. Spirulina and horseradish extracts did not show significant differences in any of the evaluated parameters, except for TFC.

### 3.2. Identification of the Optimal Supplement Dose in the Freezing Extender

**Figure 2:**
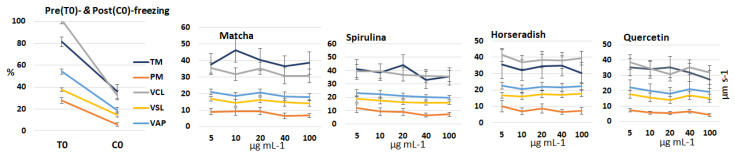
Mean (±SEM) values of sperm kinematic parameters, including total and progressive motility (TM and PM, %), curvilinear and straight-line velocity (VCL and VSL, µm s^−1^), and average path velocity (VAP, µm s^−1^), measured in sperm samples (*n* = 11) collected from seven stallions before (T0) and after cryopreservation. Samples were frozen using an extender supplemented with increasing concentrations of matcha, spirulina, and horseradish extracts, and quercetin as well as a control group without supplementation (C0).

Frozen-thawed sperm samples cryopreserved with increasing concentrations of each extract or quercetin, as well as pre-freezing (T0) and post-freezing (C0, control group) sperm samples were evaluated for several sperm kinematic parameters. The results are presented in Figure 2. Due to the marked inter-stallion variability in response, no statistically significant differences were detected among the various supplement concentrations. Consequently, for the subsequent study, the concentration showing the best overall performance was selected for each extract and for quercetin: 10 µg mL^−1^ for matcha, and 5 µg mL^−1^ for spirulina, horseradish, and quercetin.

A preliminary comparison of the extracts and quercetin, independent of the administered dose, revealed a significant main effect of the extracts on progressive motility (*p* = 0.023). A trend toward significance was also observed for total motility (*p* = 0.084) and VCL (*p* = 0.072). However, neither the extracts nor quercetin differed significantly from the control group. With the exception of progressive motility, all parameters were significantly influenced by individual stallions (*p* = 0.001).

### 3.3. Effect of Natural Extracts and Quercetin on Various Sperm Functional Parameters

Table 2 presents the mean values of semen and sperm parameters in stallions before cryopreservation. Significant inter-stallion differences were observed for total sperm motility, VCL, lipid peroxidation, and ROS content. The effect of stallion age on fresh spermatozoa was evident only in lipid peroxidation levels.

Table 3 reports the mean post-thaw values of kinematic, bioenergetic, and oxidative/nitrosative stress parameters in sperm samples treated with freezing extenders supplemented with matcha, spirulina, horseradish, or quercetin, as well as in the unsupplemented control group. No significant group differences were observed for any of the analyzed parameters, except for MMP, which differed between spermatozoa frozen with spirulina and those frozen with quercetin. With the exception of NO levels, all parameters exhibited significant inter-stallion (*p* < 0.01) and age-related (*p* < 0.05) variability.

**Figure 3:**
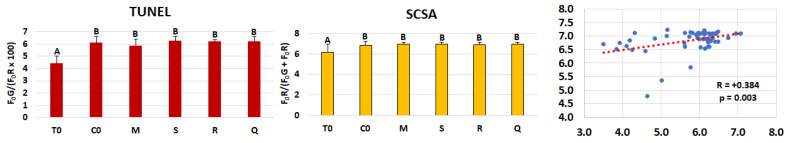
DNA fragmentation index assessed using TUNEL and SCSA, and correlation between these two tests in stallion sperm samples (*n* = 11 collected from 7 stallions) evaluated before freezing (T0) and after freezing with extenders supplemented with matcha (M), spirulina (S), horseradish (R) extracts, quercetin (Q), or without supplementation (C0). A,B (*p* ≤ 0.01).

The DNA fragmentation indices (DFI) in sperm treated with the extracts, quercetin, and the control group, including the pre-freezing spermatozoa, are shown in Figure 3. Both the direct assessment of DNA fragmentation using the TUNEL assay and the indirect evaluation of chromatin susceptibility to denaturation using the SCSA test revealed no significant differences among groups after cryopreservation, including the control. Conversely, significantly lower DFI values were observed in pre-freezing samples with both assays. Moreover, a positive and significant correlation was found between the results obtained by the two methods.

For age-related analyses, stallions were divided into two groups (≤9 years and >9 years), according to the classification proposed by Aurich et al. [58]. Fresh semen traits in stallions of these groups are reported in Table S1. Table 4 summarizes the overall results in frozen-thawed spermatozoa, assessing the main effects of treatment, stallion, age, and the age × treatment interaction. Significant age effects were detected for all parameters except DFI. Supplementation with extracts or quercetin significantly influenced only MMP. Stallion main effect was significant for all parameters except NO levels and DFI^TUNEL^, while no age × treatment interactions were observed for any variable.

**Figure 4:**
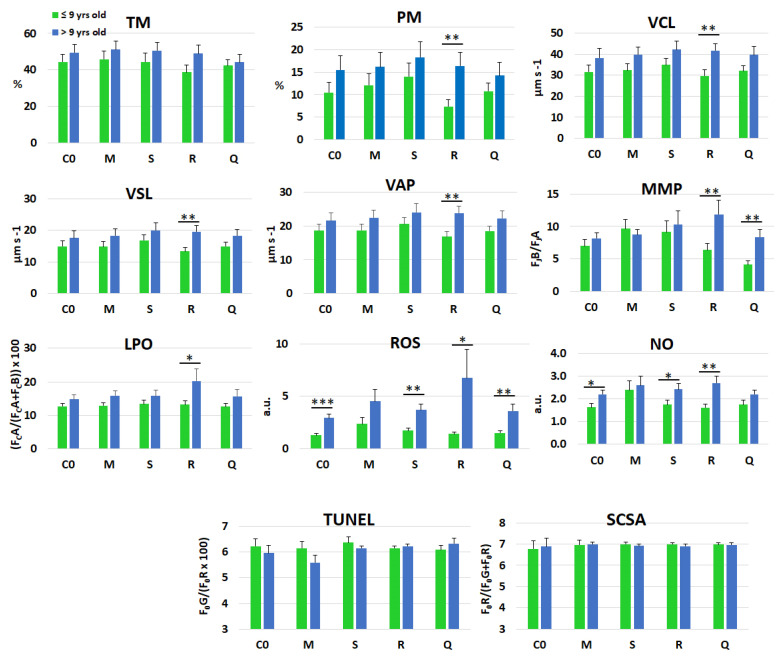
Overall mean (±SEM) values of selected kinematic—total motility (TM), progressive motility (PM), curvilinear velocity (VCL), straight-line velocity (VSL), and average path velocity (VAP)—bioenergetic—mitochondrial membrane potential (MMP)—oxidative/nitrosative stress markers—lipid peroxidation (LPO), reactive oxygen species (ROS), and nitric oxide (NO)—and DNA fragmentation index, evaluated by either TUNEL or SCSA tests, in frozen–thawed spermatozoa, following supplementation of the freezing extender with matcha (M), spirulina (S), or horseradish (R) extracts, quercetin (Q), or without supplementation (C0). Stallions (*n* = 10) were sub-grouped by age: ≤9 years (*n* = 5) and >9 years (*n* = 5). * (*p* ≤ 0.05); ** (*p* ≤ 0.01); *** (*p* ≤ 0.001).

The response of spermatozoa to treatments across age groups is illustrated in Figure 4, highlighting significant age-related differences in the horseradish treatment for PM, VCL, VSL, VAP, MMP, LPO, ROS, and NO. Age-related differences were also evident in response to quercetin for MMP and ROS levels. The latter differed between the two age groups for all treatments except matcha, and the same pattern was observed in the control group. Similarly, NO levels showed marked age-related differences across treatments, particularly with spirulina and horseradish, as well as in the control. Finally, no differences were detected between the two age groups for DFI, regardless of whether it was assessed by direct or indirect methods.

**Figure 5:**
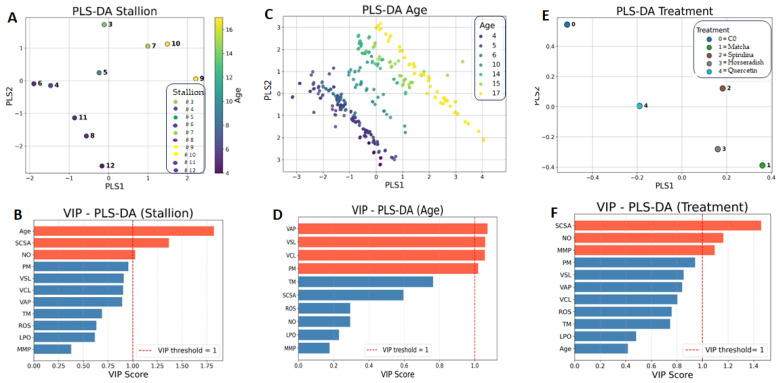
Multivariate analysis of physiological parameters in stallion sperm cryopreserved with extenders supplemented with natural extracts (matcha, spirulina, and horseradish), quercetin, or without supplementation (C0). Partial Least Squares Discriminant Analysis (PLS-DA) was used to integrate nominal variables (stallion and treatment) and the ordinal variable (age), displaying their distribution along Cartesian axes (panels (**A**,**C**,**E**)), with corresponding Variable Importance in Projection (VIP) scores shown in panels (**B**,**D**,**F**).

In the PLS-DA analysis, the variances associated with stallions, age, and treatments were clearly separated along the Cartesian axes, resulting in distinct clustering patterns (Figure 5). Interestingly, while most sperm kinematic parameters were fully involved as VIPs in the multivariate analysis focused on stallion age, both inter-stallion and treatment variability identified sperm DNA fragmentation (assessed by SCSA) and NO concentration as key VIPs. In contrast, mitochondrial activity appeared to be primarily implicated as a discriminant variable only in the comparison between treatments.

## 4. Discussion

The supplementation of cryopreservation media with extracts derived from different nutraceutical sources, each characterized by unique bioactive profiles but sharing a common antioxidant potential, together with a well-established antioxidant compound (quercetin), did not result in an overall improvement in the average freezability of equine semen. Despite the distinct phytochemical compositions of the tested products, their addition failed to consistently affect post-thaw sperm kinetics, bioenergetics, oxidative and nitrosative stress markers and DNA fragmentation index. This outcome likely reflects the intrinsic variability that typifies stallion semen freezability, which is known to vary markedly among individuals and to be influenced by multiple physiological factors, including age. Indeed, in the present study, both pronounced inter-individual variability and significant age-related differences were observed, likely obscuring any potential treatment effects. Even after stratifying stallions into age classes to control for age-related influences, treatment-related differences remained statistically nonsignificant. Nevertheless, among all the tested substances, the horseradish extract uniquely exhibited age-dependent effects, influencing most sperm parameters except total motility. Moreover, variations in reactive oxygen and nitrogen species (RONS) levels between younger and older stallions further confirmed the importance of age as a determinant of oxidative status and cryotolerance.

The lack of a general improvement following antioxidant supplementation underscores the complexity of oxidative stress dynamics during cryopreservation. Although the tested extracts shared a strong antioxidant potential, the redox environment of stallion semen may be less responsive to exogenous antioxidant inputs than that of other species [22,59,60]. In stallions, seminal plasma naturally exhibits high levels of antioxidant enzymes, such as superoxide dismutase (SOD) and glutathione peroxidase (GPx) [7], and its removal during cryopreservation may differentially affect sperm from individuals with varying intrinsic antioxidant capacities [61,62]. Consequently, supplementation with exogenous antioxidants may provide benefits only to stallions with inherently low seminal antioxidant levels, whereas those with sufficient endogenous protection may experience little or no improvement. This hypothesis aligns with previous reports demonstrating that stallion identity alone can account for up to 70% of the total variance in post-thaw semen quality traits [58], but it must also contend with the low repeatability observed for many parameters associated with seminal and sperm quality in stallions [53]. The relationships between oxidative status and DFI can also be interpreted within this framework, as no differences in DFI were observed among treatments, consistent with the results obtained for the other sperm parameters. However, in the multivariate analysis, the SCSA value emerged as one of the main VIPs when inter-stallion or treatment-related variance was considered, whereas it showed a low VIP score in relation to stallion age.

Among the tested extracts, matcha (*Camellia sinensis*) showed no consistent improvement in semen quality. The effects of *C. sinensis* supplementation during sperm cryopreservation appear to be highly species- and dose-dependent. In boars, green tea extract failed to improve motility, viability, or acrosome integrity, though it consistently reduced lipid peroxidation [29]. Conversely, in rams, supplementation with 10 µg mL^−1^ *C. sinensis* extract enhanced sperm viability, plasma membrane integrity, mitochondrial activity, and total antioxidant capacity (TAC) while reducing apoptosis and malondialdehyde (MDA) levels [30]. Similar improvements were reported in Achai bulls, where 0.75% green tea extract increased motility, viability, and membrane integrity [31]. In humans, *C. sinensis* extract improved sperm viability and mitochondrial membrane potential (MMP) and reduced ROS and DNA fragmentation at concentrations ≥ 40 µg mL^−1^, while lower doses had no effect [32]. The variable efficacy of *C. sinensis* across species likely reflects differences in sperm membrane composition, metabolic activity, and antioxidant thresholds. Moreover, the presence of caffeine, a methylxanthine known to influence sperm motility, has been proposed as a contributing factor to some of these effects [32]. However, caffeine alone has not been shown to enhance post-thaw sperm motility in stallions [63], and both caffeine and pentoxifylline have been reported to cause transient motility increases accompanied by deteriorated sperm morphology and membrane integrity [64]. Therefore, the beneficial actions of matcha are probably mediated by its catechins, polyphenols, and other antioxidant constituents rather than caffeine itself. In the current study, although 10 µg mL^−1^ matcha extract tended to improve certain parameters, the effects did not reach statistical significance due to the high inter-individual variability observed.

The extender supplementation with Spirulina (*Arthrospira platensis*) extract likewise did not lead to measurable improvements in post-thaw sperm quality. This result contrasts with several studies reporting beneficial effects in other species and even in stallions, although the outcomes have been inconsistent. In buffalo bulls, Spirulina extract at 10 µg mL^−1^ improved post-thaw motility and reduced acrosomal damage and lipid peroxidation [35]. In Arabian stallions, Spirulina supplementation during cooling did not alter sperm quality or antioxidant status [36], yet when added to the freezing extender as powder at 60 µg mL^−1^, it enhanced sperm kinetics and TAC and increased SOD and glutathione reductase (GSR) activities while reducing lipid peroxidation [36]. The discrepancies among studies may be due to differences in the preparation and chemical composition of Spirulina formulations. The present study employed an aqueous extract of dehydrated cells, whereas other studies used crude powder, which may preserve a broader range of bioactive molecules, including phycocyanin and carotenoids. Additionally, dose- and species-specific differences may contribute to the lack of significant effects. Despite testing a range of concentrations (5–100 µg mL^−1^), no treatment yielded consistent improvements, suggesting that the antioxidant activity of Spirulina may depend strongly on extraction method, compound stability, and sperm susceptibility to oxidative stress.

Horseradish (*Armoracia rusticana*) extract was evaluated here for the first time in the context of stallion semen cryopreservation. Although *A. rusticana* is known for its rich profile of glucosinolates, isothiocyanates, and phenolic compounds with reported antioxidant, antibacterial, anti-inflammatory, and even anticancer properties [65], its supplementation did not reduce lipid peroxidation or ROS generation. Overall, horseradish proved to be the least effective treatment in comparison with the other extracts and quercetin. Interestingly, the influence of *A. rusticana* became evident only when stallions were stratified by age, suggesting an interaction between the biological state of the animal and the metabolic response to supplementation. The limited efficacy of *A. rusticana* may also relate to its specific phytochemical composition, as its major antioxidant molecules may not be stable or bioavailable in the aqueous medium used for semen dilution. It is important to clarify that *A. rusticana* should not be confused with *Moringa oleifera*, sometimes referred to as the “horseradish tree.” While *M. oleifera* extracts have demonstrated cryoprotective effects on sperm in several species [60,66], the two plants differ markedly in their phytochemical and biological properties, and the current findings do not support comparable functionality for *A. rusticana* in stallions.

Quercetin was included as a positive control due to its extensively documented antioxidant activity. Nonetheless, its supplementation did not improve post-thaw sperm quality, mirroring previous inconsistent findings in stallion semen [67,68,69]. Quercetin’s effects are strongly concentration-dependent: at moderate doses (0.1–0.15 mM), it can scavenge free radicals and stabilize membranes, whereas at higher concentrations (≥0.2 mM), it may shift toward pro-oxidant behavior, compromising sperm viability and membrane integrity [67]. The concentrations used in the present study (5–100 µg mL^−1^; 0.0167–0.334 mM) encompass both potentially beneficial and deleterious ranges. Even though doses near the effective range reported elsewhere (40 µg mL^−1^; 0.134 mM) were tested, no significant effects were observed. Similar inconsistencies have been reported in other species: in boars, 50 µM quercetin improved total motility and mitochondrial function but not other kinematic parameters [59], while in humans, the same concentration enhanced post-thaw motility and DNA integrity but did not affect apoptosis [70]. Collectively, these findings suggest that quercetin’s impact depends not only on concentration but also on the species-specific oxidative environment and the composition of the extender used.

Analysis of DNA fragmentation provided further insights into the mechanisms underlying cryodamage and the limited efficacy of the tested supplements. As expected, the DNA fragmentation index (DFI) increased after cryopreservation, but no significant differences were observed among treatments. Both direct (TUNEL) and indirect (SCSA) assays yielded consistent results, showing a high degree of correlation, consistent with previous human and equine studies [55,71]. Interestingly, no age-related differences were detected, indicating that oxidative stress during cryopreservation affects sperm DNA independently of age. Notably, while inter-stallion variability in DFI was minimal in fresh semen, it became pronounced after thawing, especially in the SCSA, highlighting the intrinsic differences in chromatin stability and DNA repair capacity among individuals [72]. Moreover, in the multivariate PLS-DA analyses, SCSA emerged as a variable of importance in projection (VIP), reinforcing its role as a key determinant of cryotolerance and individual responsiveness to antioxidant supplementation.

The influence of stallion age on semen quality is well established, with older stallions often exhibiting lower motility and membrane integrity in fresh semen [73]. The present findings extend these observations to cryopreserved semen, confirming that age also modulates post-thaw sperm quality [53,71]. However, the persistence of high inter-individual variability after controlling for age indicates that chronological age alone cannot explain differences in freezability. Since all stallions in this study were maintained under uniform nutritional and environmental conditions, neither diet nor management may account for the observed variability. Instead, differences in intrinsic factors, such as sperm membrane lipid composition, mitochondrial function, and seminal antioxidant profile, likely underlie the variation in cryotolerance. These factors may interact differently with exogenous antioxidants, resulting in highly individualized responses to supplementation.

## 5. Conclusions

Overall, the findings of this study demonstrate that the addition of nutraceutical-derived antioxidants or quercetin to the freezing extender does not yield consistent improvements in post-thaw sperm quality in stallions. The considerable individual variability in cryotolerance appears to overshadow any modest treatment effects. Therefore, rather than relying on generic antioxidant supplementation, future research should focus on characterizing each stallion’s specific oxidative and metabolic profile to guide the development of personalized freezing protocols. Tailored extenders, optimized for each individual’s intrinsic cryotolerance and antioxidant balance, may represent the most promising approach for improving the efficiency of equine semen cryopreservation.

## Figures and Tables

**Table 1:** Biochemical and phytochemical evaluation of antioxidant activity and related properties in aqueous extracts of matcha, spirulina, and horseradish, compared with quercetin (as a reference for TFC) and ascorbic acid (used as a standard in selected assays).

	Matcha	Spirulina	Horseradish	Quercetin	Ascorbic Acid
	Mean ± SD	Mean ± SD	Mean ± SD	Mean ± SD	Mean ± SD
TAC	2186.7 ± 102.5 ^A^	117.6 ± 17.4 ^B^	139.8 ± 15.4 ^B^	877.8 ± 39.4 ^C^	1492.8 ± 90.6 ^D^
TRP	334.0 ± 37.7 ^A^	43.6 ± 6.9 ^B^	57.3 ± 8.1 ^B^	772.4 ± 61.4 ^C^	-
FSRA	45.7 ± 4.1 ^A^	8.6 ± 0.8 ^B^	10.5 ± 1.1 ^B^	50.9 ± 6.6 ^A^	48.6 ± 6.7 ^A^
TPC	192.7 ± 15.0 ^A^	24.3 ± 2.7 ^B^	19.6 ± 3.0 ^B^	505.5 ± 44.8 ^C^	-
TFC	282.9 ± 16.9 ^A^	46.0 ± 7.3 ^Ba^	14.9 ± 2.3 ^Bb^	-	-

TAC: total antioxidant capacity (μg ISE mg^−1^), TRP: total reducing power (μg AAE mg^−1^), FRSA: free radical scavenging activity (% of DPPH inhibition), TPC: total polyphenol content (μg GAE mg^−1^), and TFC: total flavonoid content (μg QE mg^−1^). A,B,C,D (*p* ≤ 0.01), a,b (*p* ≤ 0.05).

**Table 2:** Mean (±SD) values and their 75% confidence interval of selected parameters in fresh semen, including gel-free semen volume, sperm concentration, total sperm count, and physiological characteristics, such as sperm kinetics, bioenergetics, and oxidative/nitrosative stress markers. The significance levels of the individual and age main effects on these parameters were also reported.

		Mean ± SD	75% Confidence Interval	Inter-Stallion Variability (*p*=)	Age Variability (*p*=)
Gel-free volume	mL	48.9 ± 26.5	43.7 ÷ 54.0	0.231	0.158
Sperm concentration	×10^6^	263 ± 172	269 ÷ 331	0.213	0.960
Spermatozoa per ejaculate	×10^9^	13.6 ± 10.7	11.5 ÷ 15.7	0.343	0.587
TM	%	85.3 ± 11.0	83.1 ÷ 87.4	0.001 ***	0.575
PM	%	34.9 ± 10.7	32.8 ÷ 37.0	0.200	0.706
VCL	µm s^−1^	90.4 ± 17.1	87.1 ÷ 93.7	0.031 *	0.950
VSL	µm s^−1^	35.9 ± 7.3	34.5 ÷ 37.3	0.646	0.822
VAP	µm s^−1^	48.7 ± 9.5	46.9 ÷ 50.6	0.412	0.891
MMP	J_0_B/J_0_A	13.6 ± 9.0	12.2 ÷ 15.0	0.288	0.945
LPO	C_0_A/(C_0_A + C_0_B)	11.8 ± 4.0	11.2 ÷ 12.5	0.012 **	0.009 **
ROS content	a.u.	2.6 ± 1.0	2.4 ÷ 2.8	0.005 **	0.359
NO content	a.u.	2.5 ± 0.8	2.3 ÷ 2.6	0.287	0.471

Total motility (TM), progressive motility (PM), curvilinear velocity (VCL), straight-line velocity (VSL), average path velocity (VAP), mitochondrial membrane potential (MMP), lipid peroxidation (LPO), reactive oxygen species (ROS), and nitric oxide (NO). MMP was calculated as the ratio of the second (~595 nm) to the first (~535 nm) fluorescence intensity peak (J0B/J0A). LPO was calculated as the ratio of the first (~520 nm) fluorescence intensity peak to the sum of the first (~520 nm) and second (~590 nm) fluorescence intensity peaks, expressed as C0A/(C0A + C0B). a.u.: arbitrary units of fluorescence. * (*p* ≤ 0.05), ** (*p* ≤ 0.01), *** (*p* ≤ 0.01).

**Table 3:** Mean (±SD) values of selected kinematic, bioenergetic, and oxidative/nitrosative stress markers in frozen–thawed spermatozoa from ten stallions, following supplementation of the freezing extender with matcha, spirulina, or horseradish extracts, quercetin, or without supplementation (C0).

		C0	Matcha	Spirulina	Horseradish	Quercetin
		Mean ± SD	Mean ± SD	Mean ± SD	Mean ± SD	Mean ± SD
TM	%	47.0 ± 17.8	48.6 ± 18.8	47.6 ± 18.7	44.1 ± 18.2	43.3 ± 15.8
PM	%	13.1 ± 11.4	14.3 ± 11.9	16.3 ± 13.4	12.1 ± 10.7	12.6 ± 10.1
VCL	µm s^−1^	35.0 ± 16.2	36.2 ± 14.2	38.7 ± 15.3	36.0 ± 13.3	36.0 ± 13.7
VSL	µm s^−1^	16.4 ± 8.1	16.7 ± 7.7	18.5 ± 8.5	16.6 ± 7.1	16.7 ± 6.9
VAP	µm s^−1^	20.2 ± 8.9	20.7 ± 8.2	22.4 ± 9.0	20.5 ± 7.7	20.5 ± 7.5
MMP	J_0_B/J_0_A	7.6 ± 4.9	9.1 ± 5.7	9.8 ± 8.6 ^a^	9.3 ± 8.6	6.4 ± 4.8 ^b^
LPO	C_0_A/(C_0_A + C_0_B)	13.7 ± 5.8	14.4 ± 6.5	14.8 ± 7.8	15.8 ± 11.8	13.6 ± 6.6
ROS content	a.u.	2.1 ± 1.6	3.1 ± 4.0	2.6 ± 2.1	3.1 ± 6.3	2.4 ± 1.9
NO content	a.u	1.9 ± 1.0	2.5 ± 2.1	2.1 ± 1.3	2.2 ± 1.4	2.0 ± 1.0

Total motility (TM), progressive motility (PM), curvilinear velocity (VCL), straight-line velocity (VSL), average path velocity (VAP), mitochondrial membrane potential (MMP), lipid peroxidation (LPO), reactive oxygen species (ROS), and nitric oxide (NO). MMP was calculated as the ratio of the second (~595 nm) to the first (~535 nm) fluorescence intensity peak (J_0_B/J_0_A). LPO was calculated as the ratio of the first (~520 nm) fluorescence intensity peak to the sum of the first (~520 nm) and second (~590 nm) fluorescence intensity peaks, expressed as C_0_A/(C_0_A + C_0_B). a.u.: arbitrary units of fluorescence. a,b (*p* ≤ 0.05).

**Table 4:** Overall mean (±SD) values of semen variables and selected kinematic, bioenergetic, oxidative/nitrosative stress markers and DNA fragmentation index (DFI), assessed by either TUNEL or SCSA test, in frozen–thawed spermatozoa, following supplementation of the freezing extender with matcha, spirulina, or horseradish extracts, quercetin, or without supplementation (C0). Stallions (*n* = 10) were sub-grouped by age: ≤9 years (*n* = 5) and >9 years (*n* = 5). Main effects of age, extract supplementation (treatment), stallion, and the interaction between age and treatment are shown.

					Effect of		
		≤9 Years Old	>9 Years Old	Age	Treatment	Stallion	Age × Treat
		Mean ± SD	Mean ± SD	*p*=	*p*=	*p*=	*p*=
TM	%	43.1 ± 16.9	48.9 ± 18.6	0.030 *	0.623	0.001 ***	0.892
PM	%	11.0 ± 9.5	16.1 ± 12.9	0.002 **	0.436	0.001 ***	0.801
VCL	µm s^−1^	32.1 ± 12.2	40.3 ± 15.7	0.001 ***	0.795	0.001 ***	0.898
VSL	µm s^−1^	15.0 ± 6.4	18.8 ± 8.5	0.001 ***	0.681	0.001 ***	0.832
VAP	µm s^−1^	18.7 ± 6.9	22.8 ± 9.1	0.001 ***	0.711	0.001 ***	0.797
MMP	J_0_B/J_0_A	7.3 ± 6.5	9.5 ± 8.1	0.007 **	0.035 *	0.001 ***	0.081
LPO	C_0_A/(C_0_A + C_0_B)	13.0 ± 5.1	16.5 ± 11.7	0.001 ***	0.385	0.001 ***	0.543
ROS content	a.u.	1.7 ± 1.8	4.3 ± 7.3	0.001 ***	0.272	0.027 *	0.303
NO content	a.u.	1.8 ± 1.3	2.4 ± 1.5	0.001 ***	0.159	0.240	0.507
DFI ^TUNEL^	F_0_G/(F_0_R × 100)	5.13 ± 0.81	6.52 ± 0.84	0.084	0.593	0.947	0.185
DFI ^SCSA^	(F_0_R/F_0_G + F_0_R)	6.87 ± 0.49	7.00 ± 0.58	0.695	0.768	0.001 ***	0.703

Total motility (TM), progressive motility (PM), curvilinear velocity (VCL), straight-line velocity (VSL), and average path velocity (VAP), mitochondrial membrane potential (MMP), lipid peroxidation (LPO), hydrogen peroxide (H_2_O_2_), nitric oxide (NO), Terminal deoxynucleotidyl transferase (TdT) dUTP Nick-End Labeling (TUNEL), and sperm chromatin structure assay (SCSA). * (*p* ≤ 0.05); MMP was calculated as the ratio of the second (~595 nm) to the first (~535 nm) fluorescence intensity peak (J_0_B/J_0_A). LPO was calculated as the ratio of the first (~520 nm) fluorescence intensity peak to the sum of the first (~520 nm) and second (~590 nm) fluorescence intensity peaks, expressed as C_0_A/(C_0_A + C_0_B). a.u.: arbitrary units of fluorescence. DFI ^TUNEL^ was calculated as the ratio of the green emission peak (~525 nm, F_0_G) and the red emission peak (~620 nm, F_0_R) × 100. DFI ^SCSA^ was calculated as the ratio of the red emission peak (647 nm, F_0_R) to the sum of the red (647 nm, F_0_R) and the green (~530 nm, F_0_G) emission peaks. * (*p* ≤ 0.05); ** (*p* ≤ 0.01); *** (*p* ≤ 0.001).

## Data Availability

The original contributions presented in this study are included in the article/Supplementary Materials. Further inquiries can be directed to the corresponding author.

## Supplementary Materials

The following supporting information can be downloaded at: https://www.mdpi.com/article/10.3390/antiox14111363/s1, Table S1. Fresh semen traits in stallions ≤ 9 vs. >9 years (*n* = 5 per group); Figure S1. Positive controls for the TUNEL and SCSA.
