# Supplementary material for: Enhancing Stallion Semen Cryopreservation: Selected Antioxidant Extracts and Sperm Freezability
Source: Antioxidants (Basel). 2025 Nov 16;14(11):1363. doi: 10.3390/antiox14111363 (PMC12649543; doi:10.3390/antiox14111363)

Enhancing Stallion Semen Cryopreservation: Selected Antioxidant Extracts and Sperm Freezability

Raffaele Boni, Raffaella Ruggiero, Felisia De Luca, Graziano Preziosi, Maria Antonietta Ferrara, Angela Ostuni, Simone Guerriero, Alessandra Gallo, Carola Murano, and Stefano Cecchini Gualandi

Table S1. Fresh semen traits in stallions ≤ 9 *vs.* > 9 years (n = 5 per group). Mean (±SD) values of selected parameters in fresh semen, including gel-free semen volume, sperm concentration, total sperm count, and physiological characteristics, such as sperm kinetics, bioenergetics, and oxidative/nitrosative stress markers. The significance levels of the individual and age main effects on these parameters were also reported.

		≤ 9 years old<br>Mean ± SD	> 9 years old<br>Mean ± SD
Age	years	5.0 ± 0.7 **A**	14.6 ± 2.9 **B**
Gel-free volume	mL	40.6 ± 22.1	60.5 ± 27.2
Sperm concentration	x 10^6	343 ± 159 **a**	233 ± 179 **b**
Spermatozoa per ejaculate	x 10^9	13.3 ± 8.3	14.1 ± 13.6
TM	%	87.6 ± 7.7	83.2 ± 13.2
PM	%	37.8 ± 9.7	32.3 ± 11.3
VCL	μm s^{-1}	88.8 ± 16.8	91.9 ± 17.9
VSL	μm s^{-1}	34.8 ± 6.4	36.8 ± 8.0
VAP	μm s^{-1}	46.4 ± 8.5	50.8 ± 10.3
MMP	J_0B/J_0A	14.5 ± 9.3	12.7 ± 8.8
LPO	$C_0A/(C_0A+C_0B)$	12.0 ± 4.7	11.6 ± 3.3
ROS content	a.u.	2.62 ± 0.86	2.57 ± 1.17
NO content	a.u.	2.73 ± 0.85 a	2.19 ± 0.66 **b**
DFI ^{TUNEL}	$F_0G/(F_0R*100)$	4.65 ± 0.66	4.14 ± 0.62
DFI ^{SCSA}	(F_0R/F_0G+F_0R)	6.44 ± 0.85	5.86 ± 0.93

Figure S1. Positive controls for the TUNEL and SCSA assays. Fixed pre-freeze stallion sperm samples were incubated for 24 hours with 300 or 600 μM H_2O_2 before performing the DNA fragmentation assays.

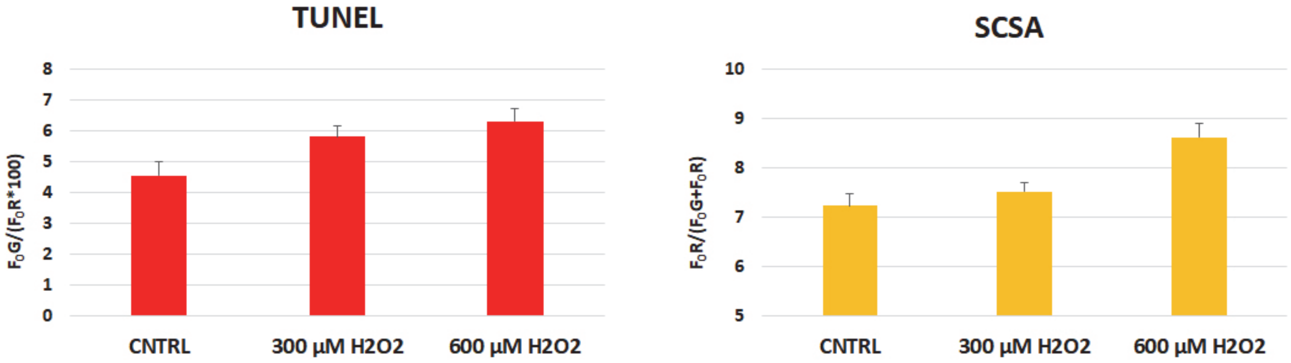

Supplement: Supplementary file 1 [file antioxidants-14-01363-s001.zip › antioxidants-3957358-supplementary.pdf]
